# Why institutional ethnography? Why now? Institutional ethnography in health professions education

**DOI:** 10.1007/s40037-019-0499-0

**Published:** 2019-02-11

**Authors:** Grainne P. Kearney, Michael K. Corman, Nigel D. Hart, Jennifer L. Johnston, Gerard J. Gormley

**Affiliations:** 1 Centre for Medical Education, Queen’s University Belfast, Belfast, UK; 2 Department of Sociology & Anthropology, and Faculty of Nursing, The University of Prince Edward Island, Charlottetown, Canada; 3 Clinical Skills Education Centre, Medical Biology Centre, Queen’s University Belfast, Belfast, UK

**Keywords:** Institutional ethnography, Health professions education, Qualitative research, Critical methodology

## Abstract

This ‘A Qualitative Space’ article takes a critical look at Dorothy Smith’s approach to inquiry known as institutional ethnography and its potentiality in contemporary health professions education research. We delve into institutional ethnography’s philosophical underpinnings, setting out the ontological shift that the researcher needs to make within this critical feminist approach. We use examples of research into frontline healthcare, into the health work of patients and into education to allow the reader to consider what an institutional ethnography research project might offer. We lay out our vision for potential growth for institutional ethnography research within the health professions education field and explain why we see this as the opportune moment to adopt institutional ethnography to meet some of the challenges facing health professions education in a way that offers informed change.

A Qualitative Space highlights research approaches that push readers and scholars deeper into qualitative methods and methodologies. Contributors to *A Qualitative Space *may: advance new ideas about qualitative methodologies, methods, and/or techniques; debate current and historical trends in qualitative research; craft and share nuanced reflections on how data collection methods should be revised or modified; reflect on the epistemological bases of qualitative research; or argue that some qualitative practices should end. Share your thoughts on Twitter using the hashtag: #aqualspace.

## Introduction

Having embraced, adopted and adapted from many long-established research disciplines including sociology, psychology and education, health professions education (HPE) research has now become a research field in its own right. This trajectory has transported HPE beyond its initial narrow positivist frame and supremacy of the biomedical model to take up increasingly more critical qualitative approaches to HPE inquiry. However, while qualitative methodologies such as discourse analysis, constructivist grounded theory and phenomenology have flourished, institutional ethnography has to date garnered little attention in this area of research. Institutional ethnography is a critical theory/methodology, with a particular focus on people’s everyday lives and how their lives are organized and coordinated by institutional forces. Use of institutional ethnography has prospered in clinical healthcare research, particularly in nursing, as well as in social work and education, bringing about useful insights and tangible change for frontline workers [1, 2]. We argue that institutional ethnography is a unique approach to inquiry that is especially suited to research in HPE.

In this article, we open a new dialogue in HPE by offering an opportunity to delve into institutional ethnography’s unique conceptual underpinnings. We do not intend this article to be an institutional ethnography ‘how to’ *per se*; for this, readers are directed to Ng et al. [3], Campbell and Gregor [4], Smith [5] and Smith [6]. Rather, we aim here to make this innovative approach to inquiry more accessible in HPE, by laying the foundations to enable readers to consider it and to further the use of institutional ethnography in the field of HPE research. As we considered this manuscript, we could see multiple, current research opportunities for institutional ethnography in modern HPE.

This discussion of institutional ethnography and its potentiality in relation to HPE is timely, especially given the context of contemporary HPE in industrialized and industrializing societies. While the HPE field is regulated differently depending on the national and political context, in addition to the discipline-specific field (which is often in isolation from other disciplines), the ties that bind HPE is where the ethos of neoliberalism and ‘new public management’ are visibly involved in reorganizing HPE [7].^1^ Neoliberalism refers to the ideology that the ‘market,’ and hence market-based solutions, is the most efficient and effective way to address public sector problems, whereby new public management is the method by which the ideology of neoliberalism is put into practice. As Griffith and Smith explain, ‘applying what has come to be called new public management has involved the adoption and adaptation of strategies and textual technologies that revolutionized corporate management during the 1980s and 1990s’ [7].

The creep of neoliberalism and new public management, for example, is evidenced by the imposition of evidence-based guidelines in the healthcare arena broadly and a focus on achieving and documenting ‘competency’ in HPE specifically. These shifts have brought new challenges to HPE, particularly in the context of powerful prevailing discourses that result in pervasive standardization throughout HPE [10] (e. g. in summative assessments) with unintended consequences such as the development of ‘tick box’ style questioning by students and the fear that such a digitized form of questioning may translate into their interactions with real patients. Exemplifying the increased presence of these discourses into the world of HPE, traditional management terms such as accountability and efficiency have become quiet murmurs or even common parlance for those involved with this work.

It is with these shifts in HPE in mind that our discussion of institutional ethnography follows. In the first section, we provide an in-depth overview of institutional ethnography, which includes key methodological and theoretical tenets of this ‘alternative’ sociology. In the second section, we provide three short analytical examples in order to provide readers with a sense of institutional ethnography in practice. This is followed by the third section whereby we discuss some potential areas where institutional ethnography can be used to explore and explicate contemporary HPE practices. We end this article with a brief conclusion.

## Institutional ethnography

For a thorough understanding of the complexities of institutional ethnography, it is necessary to know something about Dorothy Smith, contemporary sociologist and founder of this approach to inquiry. Institutional ethnography was borne out of her life’s work critiquing mainstream sociology, rejecting what she considered its inability to start in the real world or to explain the ‘bifurcation of consciousness’ that she experienced in her conflicting worlds of academia and parenthood [11]. She turned in particular to the teachings of Marx’s materialism and Garfinkel’s ethnomethodology, but also to Foucault, Mead, Bakhtin and Volosinov, and their insights on language, power and knowledge. Through her involvement in the feminist movement, she was introduced to the concept of ‘consciousness raising.’ This became key to her own sociological insights, encouraging people ‘to speak from themselves and their experience’ [11].

These early influences and experiences culminated in the development of institutional ethnography. Institutional ethnography is a critical qualitative theory/methodology that ‘starts from people’s everyday local experience and explores the translocal that is present in and organizes their everyday’ [12]. In institutional ethnography, we are interested in ‘how things work’ and ‘how they are actually put together’ as opposed to ‘what happens’ or ‘why things happen’ [11]. The emphasis is on what people do—their work broadly conceived—and what individuals say and know about their work as expert knowers and doers. This focus on work contrasts with more traditional forms of qualitative inquiry that tend to be organized by positivist tenets aimed at providing what DeVault and McCoy refer to as a ‘window on the informants’ inner experience’ (cited in [5]); it is this expert knowledge of work that provides the entry point into the inquiry [11].

By starting from this distinct position where institutional ethnography does, in people’s everyday worlds, it is also fundamental to point out that it does not, in contrast to some more conventional sociological and other research practices, start or end in theory.^2^ Starting in theory and using theory in traditional ways can result in what Smith calls the ‘14th floor effect’ [12] whereby theoretical concepts stand in for the social relations that exemplify the theory. Smith writes that in more mainstream approaches to social scientific inquiry, what actually happens on the ground—what people are doing—is objectified ‘above’ local happenings based on the theoretical frame deployed, displacing the presence of people as knowing subjects and their everyday doings [6]. In order to move beyond mainstream research that tends to begin with and end in theory—a remnant of positivist ways of thinking—institutional ethnographers begin with a ‘problematic.’ A researcher’s problematic ‘sets out a project of research and discovery that organizes the direction of investigation from the standpoint of those whose experience is its starting point’ [5]. A problematic is used in institutional ethnography to direct attention to ‘a possible set of questions,’ tensions, or puzzles that are ‘latent’ in, yet arise from, people’s everyday actualities [11].

Having begun in the everyday actualities, often through in-depth interviews, participant observations, and sometimes through a researcher’s own reflection of their everyday life, institutional ethnography then moves, to ‘investigate how their activities are coordinated. It aims to go beyond what people know, to find out what they are doing is connected with others’ doings in ways they cannot see’ [5]. Institutional ethnography orients to exploring and explicating the social relations that organize that experience in the institutional setting or settings in which they exist. It is thought that individuals participate in these sites of interface often without knowing and in a way that may not be initially obvious from their own standpoint within the institutional complex where they are situated. It is important to point out that institutions, rather than referring to buildings or organizations as such, are defined by Smith as ‘complexes embedded in the ruling relations that are organized around a distinctive function, such as education, healthcare, and so on’ [5]. ‘Keeping the institutional in view,’ as per McCoy, (cited in [6]) is not only fundamental but obligatory in institutional ethnography; indeed, it is thought that a common error by the inexperienced institutional ethnography researcher when collecting and analyzing data is for their focus to remain only on what is happening on the ground.

In order to keep the institution in view, a key defining feature of institutional ethnography is the mediating role that texts play in this coordination of peoples’ work; texts are viewed as being at the juncture between the everyday work people do and how everyday doings are organized and coordinated [4]. Whilst texts play a key role in many forms of critical qualitative research, institutional ethnography’s approach to texts is somewhat distinct. To provide some context, texts in institutional ethnography are viewed as ‘… definite forms of words, numbers or images that exist in a materially replicable form …reproduces them across time and space and among people variously situated’ [13]. In institutional ethnography, texts are never looked at in abstraction, devoid of the context in which people use them but once read or used in some way, they and the discourses embedded within them are viewed as being ‘activated.’ At this point they become active ‘constituents of social relations’ ([14], for further discussion, see [5, 15, 16]) and their ability to coordinate becomes visible. The analytical intent is to explore how lives are ‘put together’ across a multiplicity of different sites [17].

Smith’s work resulted in her bringing about an entirely alternative sociology*, the social organization of knowledge*. She feels that established sociology looks at the lives and social relations of people as if from the outside, forefronting objectivity by ignoring what people do in their everyday lives and their subjective experience of it [11].

Smith’s social organization of knowledge offers an alternative and critical way to look at individuals’ everyday lives in context; as a sociology *for* people as opposed to a sociology *of* people. Whilst the inquiry starts in the everyday world, it is imperative, in time, to establish how this world is organized socially and ‘put together’ by the external and text mediated ruling regime or what Smith calls ‘ruling relations’. Smith [16] defines these as a ‘complex of objectified social relations that organize and regulate our lives in contemporary society.’ It is this desire in institutional ethnography to explore and explicate ruling relations and how they organize and coordinate people’s everyday lives that the focus on texts becomes particularly important; texts have ‘actual presences in people’s activities and in how activities are coordinated both as local sequences of action [between people] and institutionally.’ [5]^3^.

In order to realize institutional ethnography as a sociology in itself and an approach to inquiry, a challenge is brought to the usual paradigm of traditional sociology and more mainstream ways of thinking. It requires both an ontological and an epistemological shift by the researcher. Ontology, according to Crotty ‘is concerned with what is, with the nature of existence, with the structure of reality as such’ [22]. The ontological shift that is required with institutional ethnography rejects speculative abstract theoretical explanations and moves instead towards what George Smith has described as a ‘sensuous world of people’s actual practices and activities’ [23]. Hence, in the social organization of knowledge, the inquiry begins in an embodied standpoint in the social, rather than beginning in abject theory [24] and seeks to trace or ‘map’ how peoples’ practices and activities are organized and coordinated by text-mediated modes of governance.

Connected to this discussion of ontology is the epistemology that institutional ethnography proffers. Epistemology ‘is a way of understanding and explaining how we know what we know’ [22]. The epistemological shift in institutional ethnography involves rejecting ‘objective accounts (the view-from-nowhere type) and instead practices a reflexive way of knowing the world she or he inhabits’ [23]. Institutional ethnography strives for a way of knowing that is experiential, from the inside, rather than the truth or objective or ideological. As George Smith summarizes ‘Objective knowledge is no longer ‘the truth’ [24] and he discusses how whilst the ontological shift came about from Dorothy Smith’s reading of Marx, this epistemological shift is due to the influence of her experiences through the feminist movement; hence its common description as a ‘Marxist feminist’ approach to sociology and social scientific inquiry. Taken together, institutional ethnography’s ontological and epistemological shifts positions institutional ethnography as a non-positivist approach in that it rejects the assumption that there is a knowable (real) reality and it rejects ‘causal logic’ [25] that often results in decontextualized simplistic analyses devoid of the authorial presence of the researcher.

## Institutional ethnography in practice

In the previous section, we provided readers with an overview of institutional ethnography in order to give a sense of its theoretical underpinnings and, more broadly, a sense of institutional ethnography as a project of discovery. With that said, it is important to note that institutional ethnography is still evolving, as scholars from numerous and distinct disciplines throughout the world continue to take it up, often in ongoing collaboration with Smith herself. Indeed, the decidedly political and activist roots of institutional ethnography are reflected in the social issues where it is often taken up, with the stated aim to promote social justice. This section aims to build on the previous section by providing three examples of institutional ethnography in practice. We provide short analytical accounts of three studies with a particular focus on frontline healthcare, the health work of patients and education. We do so in order to provide readers with strong exemplars of institutional ethnography and to allow readers themselves to gain a sense of what institutional ethnography has to offer in the context of HPE.

In *Managing to Nurse*, Rankin and Campbell [1] provide an inside look at healthcare reform and restructuring practices in Canada from the standpoint of nurses who work on the frontline. They do so by ethnographically exploring what nurses do—their work process broadly conceived—and how their work is organized by what they call technologies of management and governance. Such technologies, they argue, have embedded within them neoliberal and new managerial logics that aim to make healthcare, and specifically healthcare workers, more efficient, effective, and accountable. For example, they explore tracking systems of admissions, discharges, and transfers of patients that aim to provide more effective ways of utilizing hospital resources and managing hospital costs, and clinical pathways and related technologies that aim to standardize clinical practices and determine ‘appropriate’ levels of care required based on normalized understandings of patients and needs.

By ethnographically exploring the complex work of nurses and how their work is being organized and reorganized by these technologies of management and governance, Rankin and Campbell problematize ‘the apparent routineness of nurses’ work,’ work that is essential for the smooth functioning of contemporary healthcare systems, and show how such technologies have unforeseen consequences, what they refer to as ‘hidden dangers,’ that impact both frontline nurses and those they care for. Lastly, they explore and explicate how knowledge in healthcare is organized by text-mediated knowledge production processes that generate ‘official’ representation of what counts in institutional terms that while useful for the institutional complex in terms of administrative and managerial purposes is nevertheless devoid of what actually happens on the frontline [1].

More recently, Nichols et al. [26] used institutional ethnography to explore the health work—the ‘wide range of’ activities—that parents do to support the health and well-being of their child(ren) and how this family health work is organized by the institutional settings in which their work occurs. Drawing on qualitative data collected primarily through focus groups with parents from a diverse range of socioeconomic backgrounds, Nichols and colleagues shed light on how the health work of parents is mediated by a broad range of social determinants, including ‘individual, social, cultural and structural factors.’ They also show how such health work is often at ‘odds with managerial regimes’ and the biomedical model of health that discursively organizes hospital settings, despite, and perhaps in contradiction to, the pervasive focus on patient and family centred care in Canada (and elsewhere). In doing so, they provide a clearer understanding of how ‘health information, health education and healthcare interventions’ can be better designed to reflect the actual needs and lived experiences of a diversity of family types, therefore informing a critical public health.

In the context of education, Griffith and Andre-Bechely, [27] begin in the everyday world of parents and trace how ‘new institutional technologies of standardization and accountability’ are reorganizing educational settings. They focus specifically on the ‘kitchen table work’ parents do as they are drawn into the many different standardizing technologies—technologies that draw parents into ‘local, state, and national political and economic priorities’ in an attempt to help their children successfully meet different institutionally mandated academic standards. While this text-mediated work is changing the relationship between families and schools, Griffith and Andre-Bechely point out that in order to be successful in this changing relationship, many resources are needed. They conclude that despite the good intentions of such policies, many educational changes are a source of inequality, privileging some families over others, specifically those ‘that are able to release the mother’s time to support her children’s education, that are English-speaking, and that are more educated’ [27].

These brief summaries aim to provide readers some practical examples of institutional ethnography research, honouring the tradition in institutional ethnography where much of the learning around this approach to inquiry is achieved by reading and considering existing research. These studies exemplify how institutional ethnographers use theory differently than mainstream approaches to social scientific inquiry; rather than displacing and subsuming what people do in practice, theory in institutional ethnography is used to orient the ethnographer to focus on what people actually do, as they know it, live it, and experience it, but always with an analytical lens towards explicating how what they do is organized by text-mediated and regulated social organization (Smith’s ruling relations). The goal is to penetrate ‘sequentially deeper in the institutional relations in which people’s everyday lives are embedded’ [5]. As such, the ethnographies described above shed light on how the social world is put together and bring ‘into view the interface between individual lives and some set of institutional relations’ (McCoy, cited in [6]).

## Why institutional ethnography in HPE and where to go from here?

In the increasingly crowded arena of qualitative HPE research, what does institutional ethnography offer that is not already addressed by the current methods/theories open to researchers? Why would someone choose to learn this critical approach to inquiry? One of the things that sets institutional ethnography apart from other critical modes of inquiry stems from its ability to discuss *explicitly *what the situation is on the frontline and its outside organizing forces, allowing discovery of what may not be questioned or even apparent through other research modalities. The health professional world, whether in training or in practice, is rife with institutional hierarchies and regulations, making the field ripe for institutional ethnographic investigations. In this light, an area that could benefit from an institutional ethnography lens is a focus on the social organization of work, including taken-for-granted work, and work settings in HPE. It is through this exploration of both visible (institutionally recognizable) and unseen dimensions of work, and how individuals are socially organized, that institutional ethnography enables genuine and meaningful social change. Through empowering individuals to recognize their position with regards to the ruling relations, institutional ethnography then allows them to challenge these positions and to consider different approaches; institutional settings can be reformed in ways that reflect the actual work processes of those on the frontline. Ng et al. propose a further benefit of institutional ethnography; they advocate that as a research modality, it offers the opportunity to combine research into the education of health professionals with research into the practice of these health professionals, championing their undoubtable interlinkage instead of separating them [3]. Both this potential for transformation and this connection of the education/practice divide is likely to appeal to the pragmatic side of health professionals, both as educators and clinicians.

The current social, cultural and political landscape in health professions education provides multiple opportunities for exploration using institutional ethnography. The advent of new public management has resulted in a change in focus for what ‘counts’ for an organization, advocating for measurable outcomes and forefronting values such as accountability and efficiency. As previously stated, many studies using institutional ethnography have taken place in healthcare settings exploring how principles of new public management [7] have entered into and reorganized the work of frontline workers, including paramedics [2], nurses [1], care workers in long-term care facilities [28] and occupational therapists [29]. Webster and colleagues looked at the effect of a new policy that mandated a reduction in waiting times for patients in emergency departments, finding that the clinicians working there ‘perceived that efficiency was more important than education and was in fact the new definition of ‘good’ patient care’ [30]. This work is cited as an example where by a change in policy (activated through a text) organizes and regularizes practice [3] but with ‘hidden dangers’ [1]. The consequences of this appeared to be the emphasis on speedy rather than compassionate care where patients were perceived as obstructing this efficiency [3]. The current reliance on text-mediated forms and frameworks depicting the ‘competencies’ that health professionals should demonstrate would be an interesting space for HPE researchers. The authors of this article are particularly interested in looking at the dominance of standardization and accountability in the assessment processes used to deem students to be ‘competent’ in their chosen profession [31]. This interest emerged from our conflicted experiences as examiners who are also clinicians, practising daily with patients whose clinical conditions are anything but standardized.

Connected to this emerging trend of the dominance of managerial ‘buzz words’ such as accountability and efficiency has been the broader shift in healthcare towards practices based on evidence-based medicine (EBM), a key occupational competency taught throughout the health professions. EBM is increasingly being problematized by critical health scholars; while EBM is geared towards increasing quality of care and patient outcomes, such shifts in healthcare are also connected to the efficient use of resources in healthcare settings. For example, Rankin and Campbell [1] explain that the ‘use of a clinical pathway [a component of EBM] advances hospital efficiency by standardizing and streamlining the treatment regimens for patients with certain diagnoses.’

As healthcare and HPE become increasingly weighed down by policy and guidelines, the gap between such ‘evidence-based medicine’ and other forms of organizing and knowing healthcare and what actually happens in practice for health workers, health professions students and patients must be explored and explicated. This is especially important in this area because ways of knowing in healthcare are often characterized as ‘lines of fault’ between what is known institutionally and ‘systematic practices of “not knowing”’ [2, 17]. An example of this was seen in other work by Webster when she took up how the evidence base for acute stroke treatment actually played out in the day-to-day work of the medical teams implementing what was considered best practice. She concluded that this EBM discourse, (amongst others), ‘designed to improve patient care come into view as managerial tools designed to control the delivery of care’ [32]. Of interest to all working in healthcare, she contrasted the text-based EBM discourse with the tensions experienced by physicians trying to implement ‘best practice’ on the ground, in their various work situations, organized through texts in the forms of guidelines. Similar tensions were experienced by paramedic, nurses, and dispatchers in Corman’s research on the social organization of emergency medical services [2]. There are multiple examples of disjuncture between policy and practice in both healthcare and HPE where institutional ethnography could be informative not only to explore, but also to inform meaningful change. One possible future exploration where institutional ethnography could be useful is the current vogue for the use of portfolios in the training of healthcare professionals, and the disjuncture between the differing reactions they bring about in faculty as they enforce them compared with the experience of students required to complete them to achieve a license to practice; not to mention the strict bureaucratic confines they all find themselves adhering to [33].

Inevitably connected to the aforementioned areas of potential study, we posit the need to critically examine the ‘student experience’ of becoming a health professional that deploys an institutional ethnographic lens. Studying the processes of becoming a healthcare professional such as a doctor*,* nurse, paramedic, or other allied health professional requires a nuanced approach that can move beyond the dominant focus on the lived experience of students to examine how such experiences are socially organized within a broader educational and healthcare system. However, research to date tends to focus on the perceptions or experiences of HPE students, or, at least in the context of learning to doctor, explores how medical ‘culture’ or ‘medical habitus’ is instilled during (and after) medical school. Examining the social organization of HPE from the standpoint of key players, such as students and their teachers, will greatly add to HPE as the social organization of HPE has yet to be explored.

Finally, there is an increasing body of work in institutional ethnography specifically looking at the ‘health work’ that individuals do to navigate their health and healthcare (see earlier example) [6, 26, 28]. One of the authors of this article has a particular interest in the study of ‘high cost users’ and exploring how the health and social care system is organized to produce such trends. For instance, previous research in Canada has shown a consistent trend of a very small percentage of the population—often and problematically referred to as ‘frequent fliers’—accounting for the majority of public healthcare costs. Most studies to date on this topic have been driven by positivist epistemology, using primarily quantitative methodologies aimed at statistically understanding the demographics and drivers of this group. Little research to date has provided a complex understanding of this population and those who care for them. For example, Wise-Harris et al. [34] argue that, ‘the patient perspective’ of high emergency department users ‘is rarely represented’ in this domain of research. In addition, the perspective of those tasked with providing care to these supposed ‘frequent fliers’—their formal caregivers—are also left in abeyance in studies to date. There is a need to open up the unspoken realm of these individuals’ lived-experiences in accessing healthcare and social services, and begin to explicate how their experiences are organized to happen as they do. So perhaps adding to this literature on the ‘health work’ involved in being a patient can help give an authentic voice to patients and inform the training of those who will attend to patients in the future.

## Conclusion

We wrote this article in light of our desire to elucidate institutional ethnography as a theory/methodology of discovery that has much to offer HPE and to encourage its use. We have argued ‘Why institutional ethnography? Why now?’ for HPE research. More specifically, we argued that institutional ethnography is well-suited to empirically examine a variety of topics of interest to HPE researchers, ranging from what HPE looks like in practice based on a diversity of standpoints (e. g. students, teachers, etc.) to the ever-changing institutional environment of HPE and how such changes are (re)organizing the work of those on the frontlines of HPE. We also suggested a strong empirical examination of how the ‘student experience’ is socially organized and the work (broadly conceived) involved in producing ‘competent’ students who will soon become practising healthcare professionals.

In this light, we suggest institutional ethnography provides a critical and particularly informative approach to consider in moving HPE forward and encourage HPE researchers to make the ‘ontological shift’ we have described in Dorothy Smith’s innovative approach. We argue that institutional ethnography allows for a theoretically informed, albeit critical, lens to explore why there are large gaps between what is intended to happen (in policy) in HPE and what actually happens in practice. With that said, taking up institutional ethnography in HPE is likely to have its challenges as students and researchers conform to institutional requirements. Smith mentioned in conversation with us how she saw a strong potential for institutional ethnography in this domain but stated that ‘one of the things that I am concerned about is that I suspect that things like funding and the requirements of doing Theses, tends to detach the kind of connections with activism that were part of institutional ethnography’s origins, so it becomes something that is sort of ethnographic within a discipline but not related to the possibilities of making change’ [25].

Whilst we do not naively want to simplify the nuances of this very particular approach to inquiry, we will state that a less substantive background in the social sciences (all but one of this authorship are practising doctors) may mean that we are less burdened by the orthodoxy of those trained in more conventional sociological traditions. For those of us who have found our philosophical home in a less traditionally positivist paradigm, we are at ease with the ontology of institutional ethnography and reject its *alternative *sociology status. We muse that it actually seems quite intuitive to us as clinicians, reflecting our desire to maintain the subject as the subject and keep individual patients (and the students who will one day care for them) at the heart of our research, education and clinical practice.

We suggest that many cultural shifts are beginning to align which has brought institutional ethnography to the fore as an informative and potentially transformative approach to inquiry with which to tackle many of the social issues in HPE as suggested above. In a time of increasing pressures on frontline health workers with emphasis on ‘accountability’ and ‘competence,’ institutional ethnography offers informed resistance to neoliberalism, which having already infiltrated healthcare practice and the institutional settings in which that practice occurs, threatens much of what we do as health professions educators. Anyone interested in the various tenets of how social justice transpires in HPE should give institutional ethnography some informed consideration.

## Footnotes

1 These ideologies have become pervasive, or as some have noted, a ‘hegemonic hybrid’ [8, 9], throughout society. As Clarke and Newman explain [9], most ‘Civil Service’ arenas are organized by market-oriented relations of coordination and control in an attempt to achieve ‘the ‘lean state’’.

2 This is not to suggest that institutional ethnography is an atheoretical approach. Rather, it uses theory differently than most research methodologies; theory in institutional ethnography is used to keep the researcher rooted on the ground with the purpose to explore how lives are socially organized.

3 It is interesting to note that despite Smith’s unyielding criticism of many of the other qualitative research methods and institutional ethnography’s attempt to re-write sociology from the ground up, there has been relatively little critical literature on institutional ethnography (see [18–21] for a discussion of some critiques).
